# Mucinous adenocarcinoma presenting as adult colonic intussusception: A case report

**DOI:** 10.1097/MD.0000000000050820

**Published:** 2026-09-25

**Authors:** Ji Hee Han, Ra Ri Cha, Hyeon Uk Kwon, Jae Eun Kim, Yong Lee Kim, Jin Kwon Lee, Hyo Jung An, Ji Yoon Kwak, Hankyu Jeon, Sang Soo Lee, Jae Min Lee, Hyun Jin Kim

**Affiliations:** a Department of Internal Medicine, Gyeongsang National University School of Medicine, Jinju, Gyeongsangnam-do, Republic of Korea; b Department of Internal Medicine, Gyeongsang National University Changwon Hospital, Changwon, Gyeongsangnam-do, Republic of Korea; c Institute of Health Sciences, Gyeongsang National University, Jinju, Gyeongsangnam-do, Republic of Korea; d Department of Internal Medicine, Gyeongsang National University Hospital, Jinju, Gyeongsangnam-do, Republic of Korea; e Department of Surgery, Gyeongsang National University School of Medicine, Jinju, Gyeongsangnam-do, Republic of Korea; f Department of Surgery, Gyoengsang National University Changwon Hospital, Changwon, Gyeongsangnam-do, Republic of Korea; g Department of Pathology, Gyeongsang National University School of Medicine, Jinju, Gyeongsangnam-do, Republic of Korea; h Department of Pathology, Busan Paik Hospital, Inje University College of Medicine, Busan, Republic of Korea.

**Keywords:** adult intestinal obstruction, colon cancer, colonic intussusception, mucinous adenocarcinoma

## Abstract

**Rationale:**

Colonic intussusception is a rare cause of intestinal obstruction in adults, and is frequently associated with an underlying malignancy. Mucinous adenocarcinoma (MAC) is an uncommon histological subtype of colorectal cancer, characterized by abundant extracellular mucin and unfavorable biological behaviors. However, its presentation as a lead point for an intussusception is rare. Here we describe a rare case of MAC causing colonic intussusception.

**Patient concerns:**

We describe the case of a 75-year-old woman with multiple comorbidities who presented with hematochezia, abdominal distension, intermittent colicky pain, and progressive weight loss.

**Diagnoses:**

Colonoscopy revealed a large intraluminal mass in the ascending colon, while contrast-enhanced computed tomography revealed ileocecal intussusception with ischemic features.

**Interventions:**

Emergency right hemicolectomy was performed. Histopathology confirmed a moderately differentiated MAC without lymph node metastasis. Molecular analysis revealed a p.Gly13Asp KRAS (KRAS proto-oncogene, GTPase; protein change: glycine to aspartate substitution at codon 13) mutation with microsatellite stability.

**Outcomes:**

The patient’s prolonged immobility due to dementia and prior hip fracture surgery likely contributed to chronic bowel hypomotility, potentially exacerbating symptoms.

**Lessons:**

This case presents an unusual occurrence of MAC causing adult colonic intussusception, highlighting how tumor biology may synergize with host-related factors such as immobility to accelerate clinical deterioration. Early recognition using endoscopy and computed tomography and timely surgical resection is essential to optimize outcomes.

## 1. Introduction

Adult intussusception is an uncommon clinical entity, representing < 5% of all intussusception cases and only approximately 1 to 5% of bowel obstruction cases in adults.^[1]^ Unlike pediatric cases, which are often idiopathic and self-limiting, adult intussusception typically has an identifiable structural cause, most commonly a malignancy in colonic cases.^[2]^ Mucinous adenocarcinoma (MAC) is a rare histological subtype of colorectal cancer characterized by abundant extracellular mucin, a higher propensity for peritoneal dissemination, and a worse prognosis than that of conventional adenocarcinoma. Cases of adult intussusception with MAC as a lead point are exceedingly rare.^[3]^

Herein, we report a rare case of adult colonic intussusception caused by MAC in a bedridden older patient presenting with hematochezia, highlighting the importance of considering this entity in the differential diagnosis of bowel obstruction and of pursuing timely surgical intervention.

## 2. Case presentation

A 75-year-old woman with a history of diabetes mellitus, dementia, and right hip fracture surgery was transferred to the emergency department from a long-term care facility after a single episode of hematochezia yielding approximately 300 mL fresh blood. Her medications included clopidogrel, nonsteroidal anti-inflammatory drugs, donepezil, glimepiride, and a sitagliptin/metformin combination. She had experienced diarrhea in the previous month. Due to dementia and prior hip fracture surgery, the patient was unable to ambulate and was almost entirely bedridden.

At presentation, the patient complained of abdominal distension, intermittent colicky pain, mild nausea, progressive weight loss, and generalized weakness over the past several months. Her vital signs were stable (blood pressure 137/62 mm Hg, heart rate 76 bpm, respiratory rate 18 breaths/minute, temperature 36.6°C), but she appeared acutely ill. Physical examination revealed mild diffuse abdominal tenderness and distension.

Peripheral blood tests yielded the following results: hemoglobin, 13.1 g/dL; white blood cell count, 15,310/µL; and platelet count, 380,000/µL. Serum biochemistry revealed aspartate transaminase, 16 U/L; alanine transaminase, 9 U/L; albumin, 3.4 g/dL; alkaline phosphatase, 227 IU/L; gamma-glutamyl transferase, 130 U/L; lactate dehydrogenase, 184 U/L; and C-reactive protein, 1.0 mg/dL. Additional results included glycated hemoglobin A1c of 7.0%, carbohydrate antigen 19-9 of 48 U/mL, and carcinoembryonic antigen of 3.2 ng/mL. Plain chest radiography findings were unremarkable, but plain abdominal radiography demonstrated a bowel gas pattern suggestive of paralytic ileus.

Sigmoidoscopy was initially performed to identify the cause of hematochezia. Because the bowel preparation was relatively good, the scope was advanced to the ascending colon, where a large, smooth, reddish intraluminal mass with a rounded contour occupying the colonic lumen was identified. These findings were suggestive of intussusception (Fig. 1), prompting further evaluation with abdominal contrast-enhanced computed tomography (CT), which demonstrated (Fig. 2) ileocecal intussusception containing an approximately 3 cm irregularly enhancing lesion suggestive of a gastrointestinal tumor, with decreased bowel wall enhancement at the intussuscipiens, suggestive of ischemia or necrosis, and associated small bowel obstruction. Given the radiological evidence of ischemia and impending obstruction, nonoperative reduction was deemed inappropriate due to the high risk of perforation and the likelihood of an underlying malignancy. Therefore, emergency right hemicolectomy was selected as the definitive and safe therapeutic approach.

**Figure 1.**
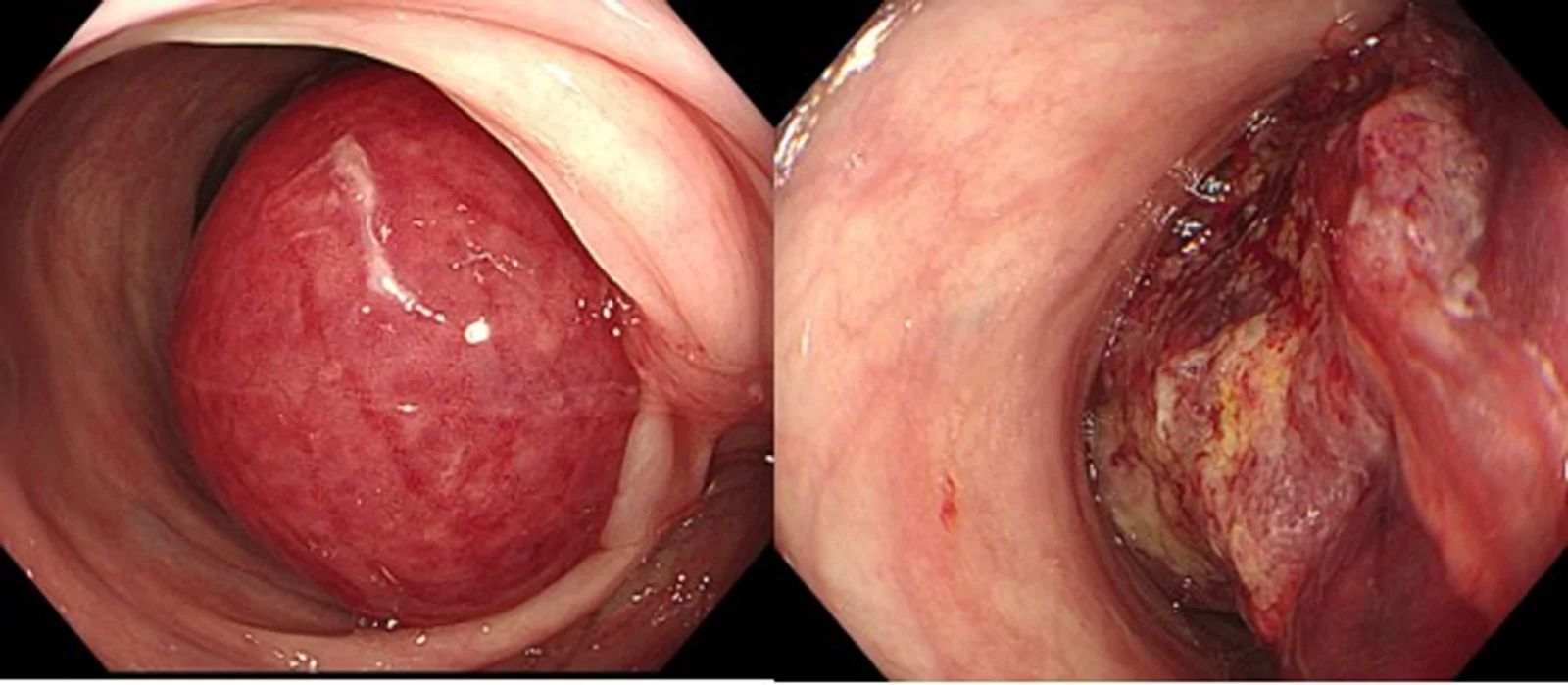
Colonoscopy findings before CT scanning showing a large, smooth, reddish, intraluminal mass with a rounded contour occupying the colonic lumen. CT = computed tomography.

**Figure 2.**
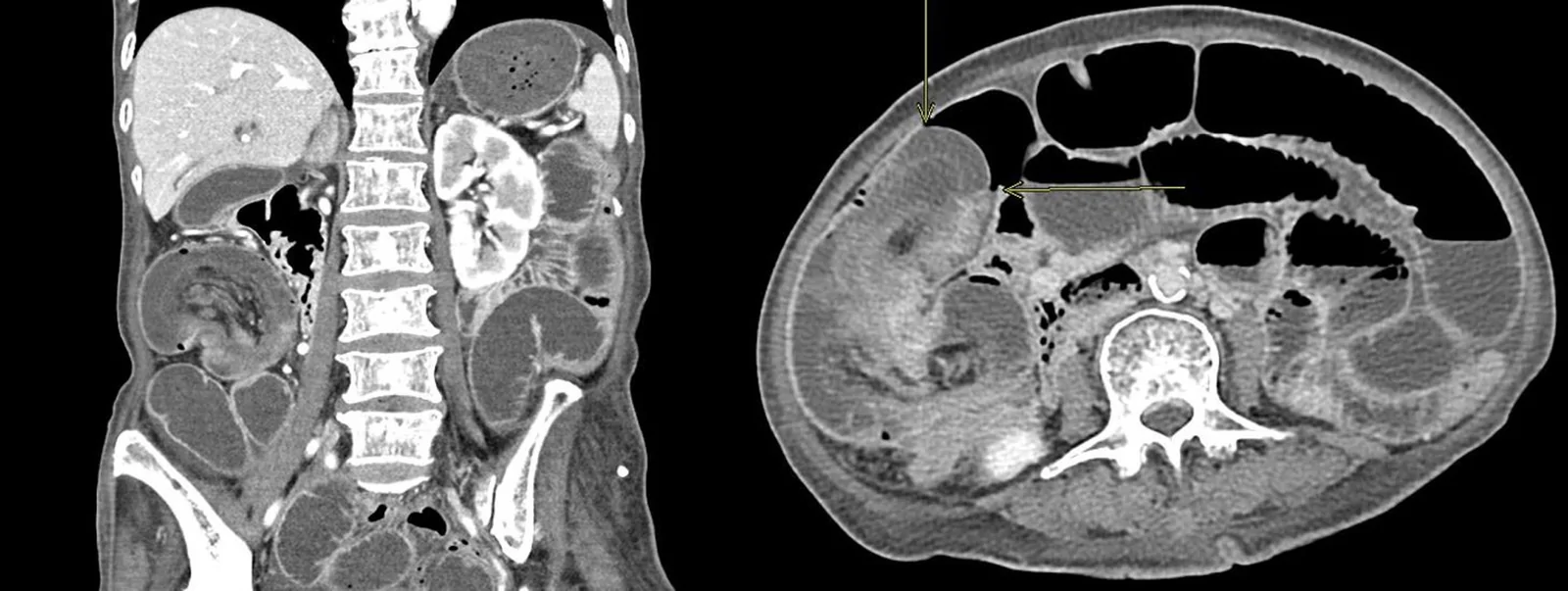
Contrast-enhanced CT scans of the abdomen taken in the (A) coronal plane (B) and axial plane, showing concentric rings suggestive of intussusception with an irregular enhancing lesion (approximately 3 cm) and decreased bowel wall enhancement at the intussusception. CT = computed tomography.

The patient underwent right hemicolectomy with side-to-side ileum–mid-transverse colon anastomosis (Fig. 3). Histopathological examination revealed a moderately differentiated MAC measuring 5.5 × 3.5 × 3 cm in the ascending colon, penetrating the visceral peritoneum but without neural, vascular, or lymphatic invasion. Resection margins were tumor-free (proximal 39 cm, distal 1 cm), and all 17 lymph nodes were negative for metastasis. Additional findings included ischemic necrosis consistent with intussusception. Extracellular mucin pools constituted more than 50% of the tumor volume, consistent with a diagnosis of MAC (Figs. 4 and 5). Immunohistochemistry showed intact nuclear expression of MLH1 (mutL homolog), and MSH2 (mutS homolog) (PMS2 [PMS2 homolog, mismatch repair endonuclease] and MSH6 were not tested) and was positive for epidermal growth factor receptor. Molecular testing showed a KRAS c.38G > A (p.Gly13Asp) mutation, with NRAS proto-oncogene, GTPase and B-Raf proto-oncogene, serine/threonine kinase V600E wild-type status. Microsatellite instability testing indicated a microsatellite stable phenotype, providing corroborating evidence against mismatch repair deficiency. The final diagnosis was ileocecal intussusception associated with a colonic MAC.

**Figure 3.**
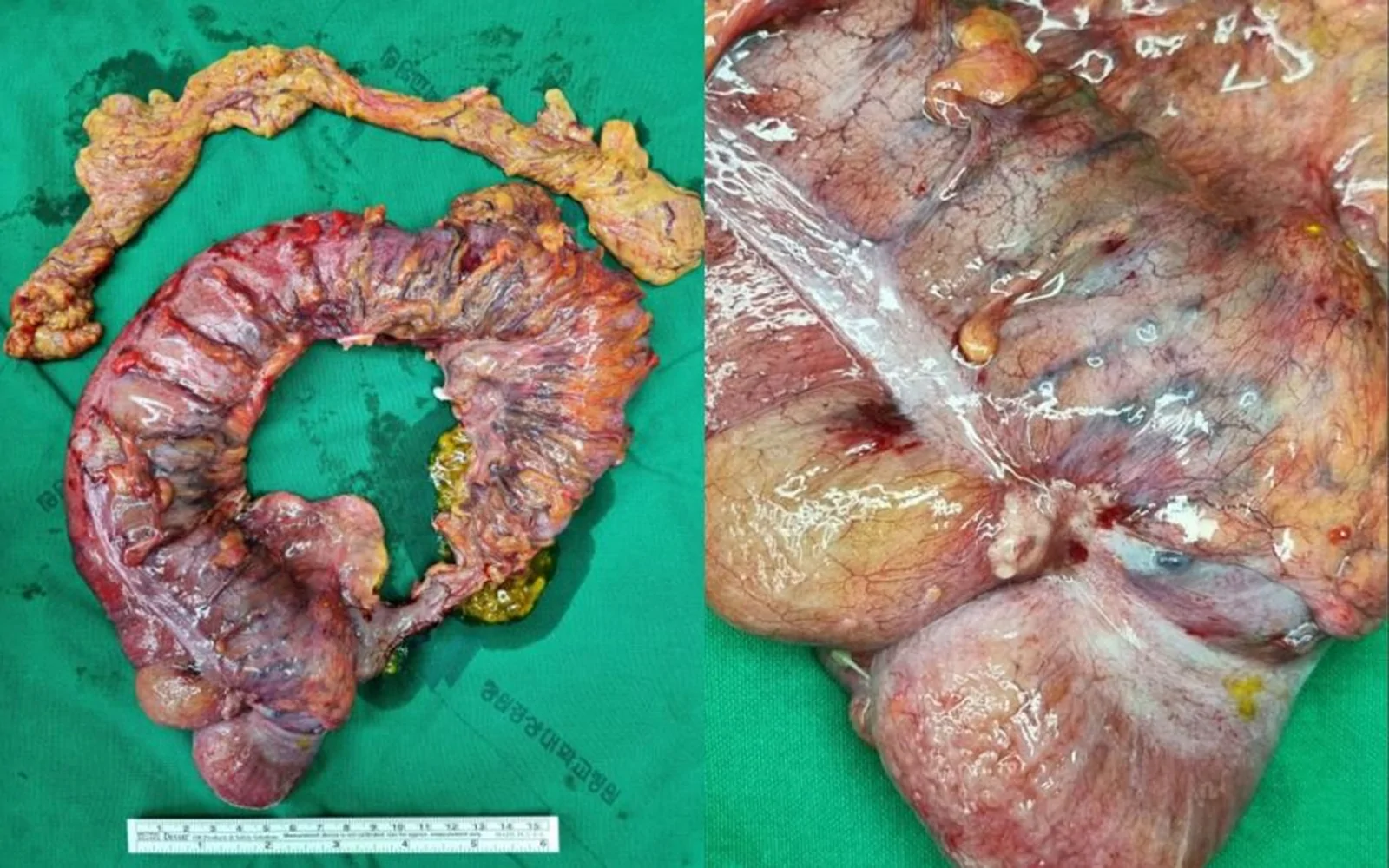
Surgical specimens after emergency surgical operation: right hemicolectomy.

**Figure 4.**
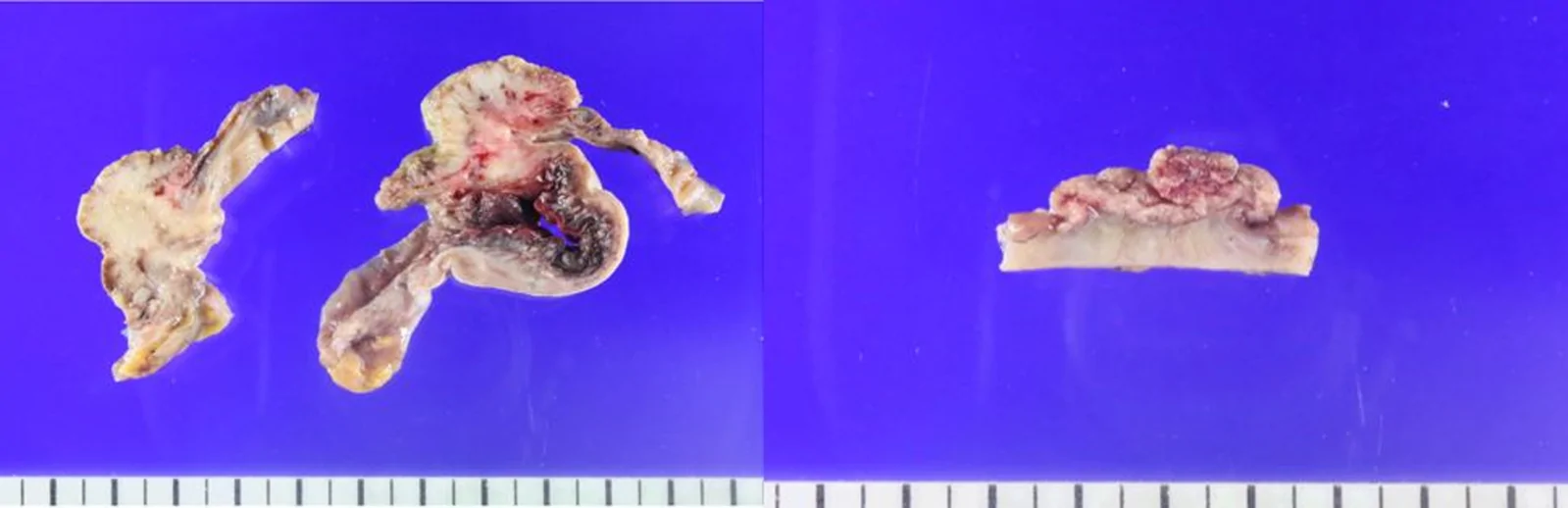
The gross specimen obtained after right hemicolectomy showing a segment of colon with an irregular, thickened, and partially ulcerated mucosal surface. The cut surface reveals a firm, grayish-white, infiltrative mass with regions of necrosis and hemorrhage, consistent with a mucinous adenocarcinoma.

**Figure 5.**
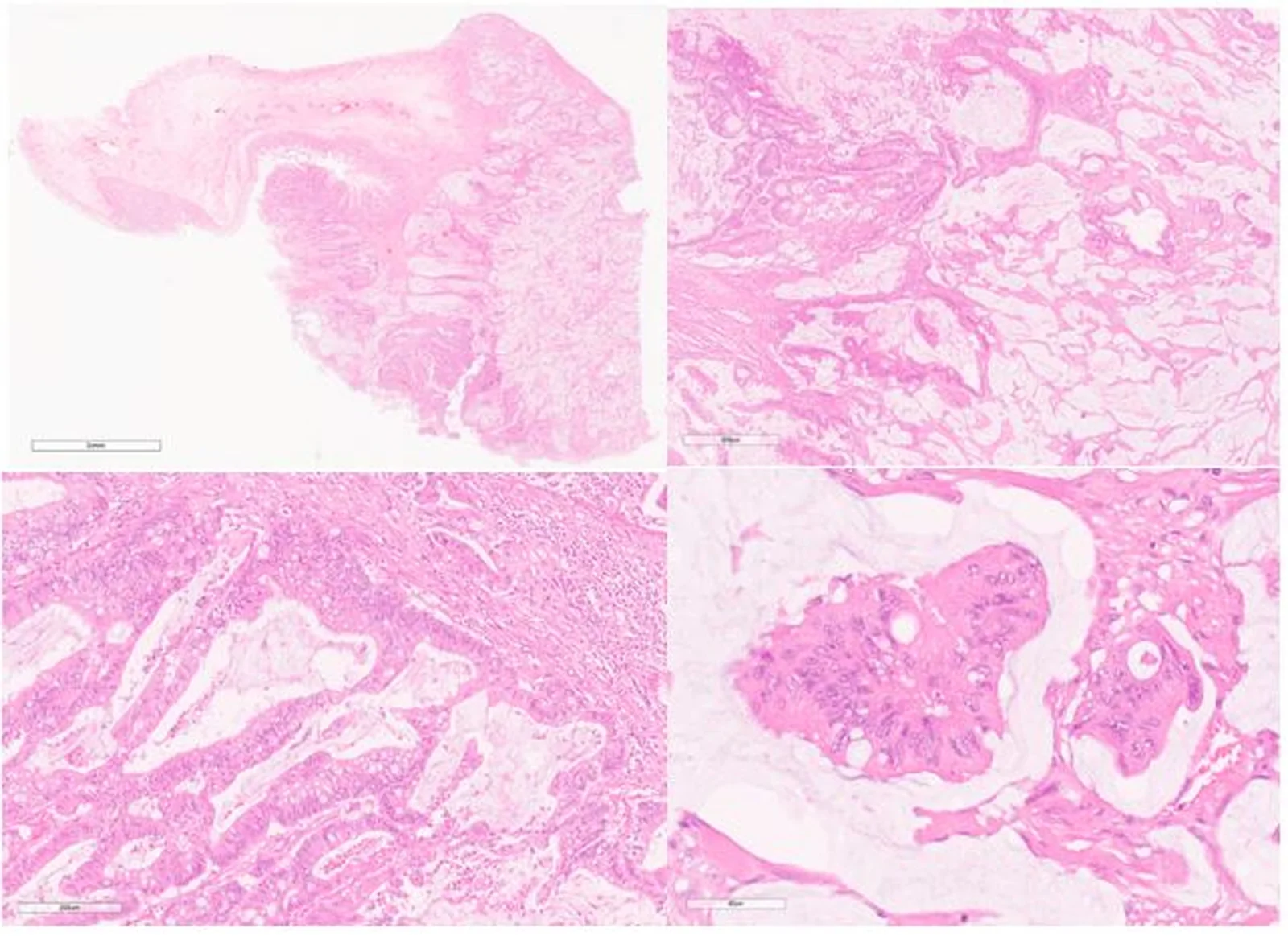
Histological analysis of surgically resected specimen. (A) A protruding mass-like lesion is observed at low magnification, comprising a central adenocarcinoma component with extracellular mucin pools on the right side (×10, hematoxylin and eosin). (B) The extracellular mucin pools constituted > 50% of the lesion, with floating glandular structures suggestive of mucinous adenocarcinoma (×40, hematoxylin and eosin). (C) The adenocarcinoma component shows high nuclear grade stratified columnar glands arranged in a back-to-back pattern (×200, hematoxylin and eosin). (D) The adenocarcinoma component shows high nuclear grade stratified columnar glands arranged in a back-to-back pattern (×200, hematoxylin and eosin).

The patient recovered uneventfully after surgery, with a gradual return of bowel function by postoperative day 4, and was discharged to a rehabilitation facility on day 21. At the 6-month follow-up, the patient remained clinically stable, without any evidence of recurrence or complications. Given the patient’s advanced age, multiple comorbidities, dementia-related functional dependency, and bedridden status, a multidisciplinary team, in consultation with the patient’s guardians, decided against adjuvant chemotherapy after weighing the marginal survival benefit against the risk of treatment-related toxicity in this frail patient.

## 3. Discussion

Adult colonic intussusception is a rare clinical entity; in most cases, an underlying structural lesion can be identified.^[4]^ Unlike pediatric cases, which are usually idiopathic and self-limiting, malignancy accounts for > 60% of adult colonic intussusceptions.^[5]^ The nonspecific presentations in adults, such as intermittent abdominal pain, altered bowel habits, hematochezia, and weight loss, commonly lead to a delayed diagnosis. Imaging modalities play a crucial role in the evaluation of adult intussusception, with abdominal CT showing high sensitivity (up to 90%) and specificity (100%).^[6]^

Endoscopy plays an important complementary role in the evaluation of intussusception in adults. Although cross-sectional imaging, particularly CT, remains the cornerstone of diagnosis, endoscopy offers the unique advantages of direct mucosal inspection and tissue sampling. This can be particularly useful in identifying a neoplastic lead point, thus clarifying the extent of luminal involvement and excluding other coexisting or mimicking pathologies, such as polyps, inflammatory masses, or ischemic changes. Furthermore, endoscopic assessment may aid in preoperative planning by characterizing lesion morphology and location, although its utility can be limited in the presence of complete obstruction or severe luminal narrowing.^[5]^

The endoscopic findings of adult intussusception typically include a smooth, rounded, intraluminal mass with mucosal erythema and edema, sometimes accompanied by central ulceration or necrosis. In the present case, sigmoidoscopy revealed a large, smooth, reddish mass with a rounded contour occupying the colonic lumen, which was consistent with these characteristic features and supported the diagnosis prior to radiological confirmation.

MAC is an uncommon histological subtype of colorectal cancer characterized by abundant extracellular mucin, a higher rate of peritoneal dissemination, and a poorer prognosis than conventional adenocarcinoma.^[7,8]^ However, it rarely functions as a lead point for an intussusception. Given the abundant extracellular mucin characteristic of these tumors, they can cause marked luminal distension, potentially disrupting normal peristalsis and facilitating telescoping of the bowel.^[1]^ Furthermore, the mucinous component may promote more rapid tumor growth and local invasion, as suggested in the present case,^[9]^ in contrast to reports in which MAC behaved in a more indolent manner.^[10]^

Beyond tumor-related mechanisms, host-related factors likely contributed to this patient’s clinical course. The patient’s prolonged bedridden state, secondary to dementia and hip fracture surgery may have induced chronic bowel hypomotility, consistent with prior evidence linking physical inactivity to functional constipation via impaired enteric neuromuscular function.^[11–13]^Pedersen et al similarly reported a high prevalence of constipation in the first 30 days after hip fracture, closely associated with immobilization.^[13]^ In the presence of a mass lesion, such motility impairment could plausibly have facilitated intussusception, underscoring the importance of considering both tumor biology and host-related functional status in frail older patients.

In cases of adult intussusception, surgical management is generally advocated because of the high incidence of underlying malignancies and the oncological principle that recurrence is inevitable if the lead point is not removed. In addition to resolving the obstruction, resection offers the critical advantages of a definitive histopathological diagnosis and appropriate staging. Simple reduction without resection may carry the risks of tumor cell dissemination, bowel perforation, and subsequent recurrence; as such, it is rarely recommended outside of highly specific circumstances. Several reviews and retrospective studies have reinforced the role of primary en bloc resection as the most reliable therapeutic approach, emphasizing that the operative strategy should be tailored to the anatomical site and the patient’s overall condition.^[14,15]^ In the present case, timely diagnosis with colonoscopy and CT allowed for prompt surgical planning, avoiding any further ischemic damage. The resected specimen revealed no lymph node metastasis, which is a favorable prognostic factor; however, given the aggressive nature of MAC, close postoperative surveillance is warranted. A limitation of this case is that mismatch repair immunohistochemistry was restricted to MLH1 and MSH2, without PMS2 or MSH6 staining; however, the concordant microsatellite stable result on microsatellite instability testing provides corroborating evidence against mismatch repair deficiency in this tumor.

Compared with previous reports, this case highlights the following 3 important points: MAC can present with a more rapid clinical course when combined with predisposing host factors, early endoscopic and radiologic evaluation is essential among older patients with nonspecific abdominal symptoms, and multidisciplinary decision-making remains critical in optimizing outcomes. Clinicians should maintain a high index of suspicion for rare causes of intussusception in adults, particularly in frail or immobile patients, to ensure timely diagnosis and treatment.

## 4. Conclusion

This case highlights the rare but important role of MAC as a lead point of colonic intussusception in adults. Early recognition, supported by targeted imaging and timely surgical intervention, is essential to optimize outcomes. Integrating tumor biology with patient-specific clinical factors is critical for achieving an accurate diagnosis, individualized management, and improved prognosis.

## Author contributions

**Conceptualization:** Ji Hee Han.

**Investigation:** Yong Lee Kim, Ji Yoon Kwak.

**Methodology:** Hyeon Uk Kwon.

**Resources:** Jin Kwon Lee, Hyo Jung An, Jae Min Lee.

**Supervision:** Jae Eun Kim, Hankyu Jeon, Hyun Jin Kim.

**Visualization:** Sang Soo Lee.

**Writing – original draft:** Ji Hee Han.

**Writing – review & editing:** Ra Ri Cha.
